# Validity and Reliability of the activPAL4^TM^ for Measurement of Body Postures and Stepping Activity in 6–12-Year-Old Children

**DOI:** 10.3390/s23094555

**Published:** 2023-05-08

**Authors:** Esraa Burahmah, Sivaramkumar Shanmugam, Daniel Williams, Ben Stansfield

**Affiliations:** School of Health and Life Sciences, Glasgow Caledonian University, Cowcaddens Road, Glasgow G4 0BA, UK; esraa.burahmah@gcu.ac.uk (E.B.); sivaram.shanmugam@gcu.ac.uk (S.S.); dwilli212@caledonian.ac.uk (D.W.)

**Keywords:** children, reliability and validity, activity monitor, posture, stepping, sedentary time, sitting time

## Abstract

A link between inappropriate physical behaviour patterns (low physical activity and high sedentary behaviour) and poor health outcomes has been observed. To provide evidence to quantify this link, it is important to have valid and reliable assessment tools. This study aimed to assess the validity and reliability of the activPAL4^TM^ monitor for distinguishing postures and measuring stepping activity of 6–12-year-old children. Thirteen children (8.5 ± 1.8 years) engaged in pre-determined standardised (12 min) and non-standardised (6 min) activities. Agreement, specificity and positive predictive value were assessed between the activPAL4^TM^ and direct observation (DO) (nearest 0.1 s). Between-activPAL4^TM^ (inter-device) and between-observer (inter-rater) reliability were determined. Detection of sitting and stepping time and forward purposeful step count were all within 5% of DO. Standing time was slightly overestimated (+10%) and fast walking/jogging steps underestimated (−20%). For non-standardised activities, activPAL4^TM^ step count matched most closely to combined backward and forward purposeful steps; however, agreement varied widely. The activPAL4^TM^ demonstrated high levels of reliability (ICC(1, 1) > 0.976), which were higher in some instances than could be achieved through direct observation (ICC(2, 1) > 0.851 for non-standardised activities). Overall, the activPAL4^TM^ recorded standardised activities well. However, further work is required to establish the exact nature of steps counted by the activPAL4^TM^.

## 1. Introduction

Physical activity (PA) has a prophylactic effect against many major non-communicable diseases [1]. Additionally, physical inactivity and sedentary behaviour (SB) have been attributed to many health problems [2,3,4]. To establish relationships between physical behaviour characteristics and health outcomes, it is necessary to be able to quantify PA and SB using a valid and reliable assessment technique. While subjective measures are available (e.g., IPAQ, GPAQ [5]) measurement using body worn monitors provides objective evidence of behaviour without associated biases of self-report.

The activPAL^TM^ family of monitors (PAL Technologies™, Glasgow, UK) are thigh-worn devices that allow the objective measurement of both PA and SB [6,7]. ActivPAL^TM^ is a research-grade piezoelectric accelerometer allowing detection of stepping activity and postural change [8]. Evidence concerning the validity of the activPAL^TM^ family of monitors has been summarised by Wu et al. and O’Brien et al. [6,7]. Generally, reports suggest that the monitors demonstrate high validity in step detection [1] and that sitting and standing are precisely detected [7]. However, limitations at both low and high speeds of stepping are highlighted. Unfortunately, neither Wu et al. [6] nor O’Brien et al. [7] reported the version of the activPAL^TM^ devices, suggesting that they view all versions of the monitor as equivalent. Validity and reliability have been reported for particular versions of the monitor for specific populations during a variety of activity protocols (e.g., for the original monitor activPAL^TM^ (adults [9], older adults [10]) and activPAL3^TM^ [11]). When compared to video-based direct observation of physical behaviour, performance under more naturalistic conditions (e.g., performance of activities of daily living where participants choose their specific movement sequences and patterns) has revealed lower levels of validity than standardised activities (e.g., [11]).

When new versions of devices are introduced, it is important to establish their validity and reliability. The activPAL4^TM^ iteration of the activPAL^TM^ has been presented as a device with hardware adaptation from previous versions of the monitor. While changes to posture and step detection are not explicitly detailed in the device documentation, it is possible that reliability and validity of the monitor have been altered compared to previous versions. Therefore, it is important to examine the monitor’s ability to measure accurately and reproducibly in various populations to ensure that the possible errors fall within an acceptable range.

Children’s physical behaviour, particularly of younger children, is generally considered to be more variable in nature than adolescents or adults. It is possible that differences in movement patterns may affect reliability and validity of monitors used to measure that activity. Assessment of devices under both standardised and more challenging conditions would provide reassurance that outcomes are reasonable representations of actual physical behaviour within this younger population.

The aim of the study was to evaluate the reliability and validity of the activPAL4^TM^ activity monitor in detecting sitting postures, upright postures and stepping activity in children aged 6–12 years old under both standardised and non-standardised conditions.

## 2. Materials and Methods

### 2.1. Participant Selection and Recruitment

A convenience sample of typically developing children between the ages of 6–12 years was recruited into the study through personal contacts of the lead author in Kuwait City, Kuwait (October 2020–August 2021). This study was conducted according to the guidelines of the Declaration of Helsinki and approved by the Ethics Committee of the School of Health and Life Sciences at Glasgow Caledonian University, August 2020, ref: HLS/PSWAHS/19/232.

The parents/guardians of potential participants were provided with study information and asked to contact the research team if the child under their care might consider taking part. After any questions had been answered, potential participants were assessed against the inclusion/exclusion criteria and written informed consent/assent taken appropriately from the parent/guardian and child by the lead author. Participants were assured that they could withdraw from the study at any point without giving reasons. Children with cognitive impairments and children who had an acute lower limb injury (excluding bruises and scrapes) were excluded from the study.

Children aged 6–12 years were chosen for this study as it was felt likely that they would perform movements that were more variable than other age groups and so present a robust challenge to the activPAL4^TM^’s ability to adequately characterise that activity. Differences in body proportions between different age groups may affect outcomes from the monitor, so it is important to assess the validity and reliability across all ages.

### 2.2. The activPAL4^TM^

A pool of 14 activPAL4^TM^ monitors was used with outcomes developed using software version PALanalysis v8.10.8.32 with settings of minimum non-upright period and minimum upright period set to 2 s [12].

**Figure 1:**
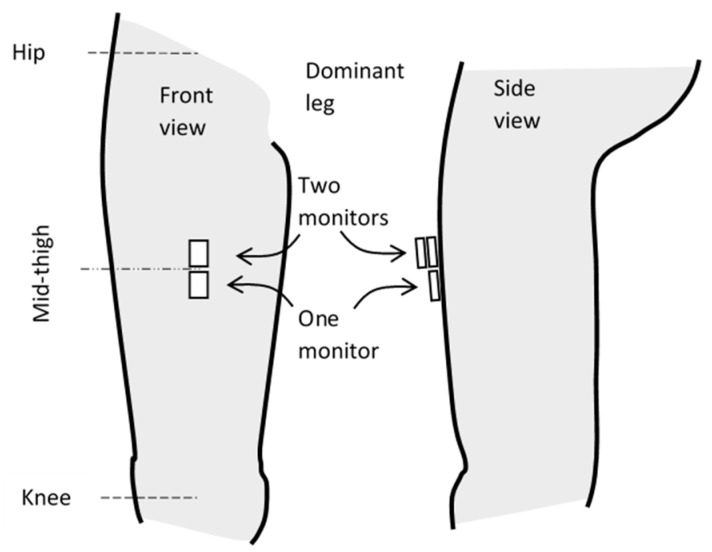
Placement of the activPAL4^TM^ monitors on the front of the thigh.

For each participant, three activPAL4^TM^ monitors were selected non-randomly from the pool of 14 and arbitrarily allocated to one of three locations. Two monitors were placed one on top of the other, with the third monitor placed just distally to these, all on the front of the thigh of the dominant leg at approximately the mid-point of the thigh (Figure 1). ActivPAL4^TM^ monitors were firmly attached to the skin by securing them with hypoallergenic tape.

A more robust test of reliability could have been achieved by allocating the activPAL4^TM^ devices randomly, as it would have ensured that each device had an equal chance of being placed in all locations. Even though the allocation of devices was non-random, the study design still allowed for a rigorous assessment of the reliability of the activPAL4^TM^.

For the current study, the event-based output of the activPAL4^TM^ proprietary software (v8.10.8.32) was used. This format reports a particular ‘bout’ of activity as an ‘event’ (to the nearest 10th of a second in duration). This would be either a period of sitting (or lying), a period of standing without stepping or a stride (2 steps). Therefore, for continuous periods of stepping activity, a sequence of stride events is reported.

### 2.3. Data Collection

The participants were video recorded, using a single video recorder, while performing a set of given tasks. Videos were recorded in MP4 format and analysed using VLC media player (open access software, version 3.0.11). Interpretation of the video recording (direct observation (DO)) of physical behaviour was considered the gold standard for this study.

Participants were asked to complete two types of activities: standardised activities (duration 12 min) and non-standardised activities (duration 6 min) (Table 1) similarly to previous validation studies [11].

The standardised and non-standardised activities chosen for this study may not fully represent real-life scenarios and, therefore, potentially limit generalizability of the findings. Nonetheless, the authors selected a wide range of activities that required various types of movements and stepping types, hoping that the findings would be applicable to real-life conditions.

#### 2.3.1. Standardised Activities

Standardised activities were selected to provide examples of sitting, standing without stepping and stepping activity in blocks of time. Standardised activities were performed in an indoor area approximately 5 × 5 m. When sitting, children were allowed to choose a preferred seat type (plastic children’s chair, adult sized hard surfaced chair, adult sized soft armchair). Activities were performed at the child’s self-selected speed. Instructions were given to sit, stand, walk at a normal self-selected speed or to run for the designated time period. Children were sometimes not cooperative, resulting in change over times between activities not being precise and delays in response to performing activities as requested. All activities had to be performed within the laboratory area, resulting in non-linear motion for stepping activities.

#### 2.3.2. Non-Standardised Activities

Non-standard activities were selected to provide examples of a variety of stepping modalities interspersed with periods of standing without stepping. Three non-standard activities were selected to provide situations in which children could perform in a self-selected manner with changes in direction, slowing down, speeding up and stepping in different directions. The three activities were ‘keep up the balloon’, ‘throw hoops over a post’ and ‘musical chairs’ (See Table 1 for full description).

### 2.4. Data Analysis

#### 2.4.1. Direct Observation Posture and Stepping Characterisation

Three raters (EB, DW, BS) independently evaluated all video records of all participants. Definitions of posture and stepping activity were used to attempt to develop a consistent interpretation of the video records (Table 2).

In order to accurately assess the reliability and validity of step detection using the activPAL4^TM^, it is necessary to define what constitutes a step. This is because different step definitions can result in different step counts, which can impact the apparent validity and reliability of the monitor. The step count definitions used in this work were pragmatically defined based on observations of movement patterns and what the authors wished to characterise as a step.

DO was used to characterise the posture (sitting or upright), stepping time and number of steps of the standardised activities. For the non-standardised activities, the movement patterns of the children were expected to be complex such that distinguishing time periods of standing from stepping would be difficult and extremely time consuming. Therefore, for the non-standardised activities, only steps taken were characterised and total steps across all three activities were analysed together. Timings were taken from the video to the nearest 10th of a second.

The average results from the three DO characterisations were used to compare with the activPAL4^TM^ results. For step count, simple means were taken. For posture changes, the mean time point of the change was utilised. Where short periods of intermediate standing posture between sitting and stepping were identified, these were included in the average DO record if 2/3 observers recorded this.

To prepare a continuous record of posture allocation from the DO record, data were interpolated across the whole time of the study to tenths of a second, which could be directly compared with activPAL4^TM^ output.

#### 2.4.2. Validity

To measure the agreement between DO and activPAL4^TM^ posture and step count, Bland–Altman plots [13] were used, with limits of agreement (LoA, ±1.96 × SD) representing measurement error. Outcomes from the single activPAL4^TM^ attached lower on the thigh were used for the agreement analysis. The percentage difference between measures (Equation (1)) was plotted against the mean of measurements (Equation (2)):

(1) $$y\;\mathrm{axis}=\frac{\mathrm{activPAL}4\;\mathrm{measure}-\mathrm{DO}\;\mathrm{measure}\;}{\mathrm{DO}\;\mathrm{measure}}\times 100$$

(2) $$x\;\mathrm{axis}=\frac{\mathrm{DO}\;\mathrm{measure}+\mathrm{activPAL}4\;\mathrm{measure}}{2}$$

An a priori upper limit of 5% difference was chosen as being acceptable to evaluate the activPAL4^TM^ outcome measurements in relation to DO.

Second-by-second analysis of postural agreement, sensitivity and positive predictive value (PPV) between DO and activPAL4^TM^ were also calculated [14].

Percentage of agreement was calculated for each participant across standardised activities to characterise the proportion of time posture classification (sitting, standing, stepping) of DO and activPAL4^TM^ were identical (Equation (3)):

(3) $$\%\;\mathrm{of}\;\mathrm{Agreement}=\frac{\mathrm{number}\;\mathrm{of}\;\mathrm{seconds}\;\mathrm{where}\;\mathrm{activPAL}\;\mathrm{posture}\;=\;\mathrm{DO}\;\mathrm{posture}\;}{\mathrm{number}\;\mathrm{of}\;\mathrm{seconds}\;}\;\times \;100$$

Within the time that a participant was observed (DO) to be in a certain posture, sensitivity was determined as the proportion of time that the activPAL4^TM^ agreed with this classification (Equation (4)):

(4) $$\mathrm{Sensitivity}=\frac{\mathrm{number}\;\mathrm{of}\;\mathrm{seconds}\;\mathrm{where}\;\mathrm{posture}\;\mathrm{classifications}\;\mathrm{of}\;\mathrm{activPAL}4\;\mathrm{and}\;\mathrm{those}\;\mathrm{of}\;\mathrm{DO}\;=\;A\;}{\mathrm{number}\;\mathrm{of}\;\mathrm{seconds}\;\mathrm{where}\;\mathrm{DO}\;\mathrm{posture}\;\mathrm{classification}\;=\;A}\;\times \;100$$

Within the time that the activPAL4^TM^ determined a particular posture, PPV characterises the proportion of this that agrees with DO (Equation (5)):

(5) $$\mathrm{PPV}=\frac{\mathrm{number}\;\mathrm{of}\;\mathrm{seconds}\;\mathrm{where}\;\mathrm{posture}\;\mathrm{classifications}\;\mathrm{of}\;\mathrm{activPAL}4\;\mathrm{and}\;\mathrm{those}\;\mathrm{of}\;\mathrm{DO}\;=\;A\;}{\mathrm{number}\;\mathrm{of}\;\mathrm{seconds}\;\mathrm{where}\;\mathrm{activPAL}4\;\mathrm{posture}\;\mathrm{classification}\;=\;A}\;\times \;100$$

#### 2.4.3. Reliability

The reliability of the activPAL4^TM^ was evaluated by examining the outcomes of the sets of three devices worn simultaneously. Additionally, to establish the reliability of the DO measurements, a comparison between the three observers’ outcomes was made.

First overall percentage agreement was calculated within monitors and observers based on the interpolated tenth of a second data for sitting, standing and stepping for the standardised activities. To interpret levels of agreement, a priori values were set: >90% as excellent, >70% as good.

To assess reliability of the activPAL4^TM^ for determining step count in standardised normal and fast walking and the non-standardised activities, the interclass correlation was calculated (ICC(1, 1)) [15]. The ICC(1, 1) was used as this best matched the assessment which used an arbitrary allocation of a monitoring device from a larger pool of devices.

To assess reliability of the DO observers for determining step counts (purposeful forward, backward and all steps) during both standardised and non-standardised activities, the ICC(2, 1) was calculated as observers were consistent but from a larger possible pool.

ICC score ratings of <0.40 were considered poor, 0.40 to 0.75 fair to good, >0.75 very good and >0.90 excellent.

## 3. Results

Fifteen healthy Kuwaiti children took part in the study. Data of two children were removed from the analysis as one of them had incomplete video recordings, and for the other, the activPAL4^TM^ was malfunctioning. Data from 13 participants (6M/7F) remained in the study (mean age 8.5 years old, SD 1.8, range 6.3–12.2). Eight of the children attended private schools and five attended public schools. The data collection from these participants took place in the summer vacation of 2021.

The sample size was relatively small due to the intensive data analysis required in DO. The sample covered the age range of interest and the activity protocol allowed a high degree of self-selection of movement patterns within the activities. Therefore, while a larger sample would have provided a more robust test of the validity and reliability of the activPAL4TM, the data recorded should have highlighted key elements of device performance.

Of the 14 activPAL4^TM^ devices, each was used between one and five times overall, with each monitor being used a maximum of four times in any one location. Across all participants, nine or 10 different monitors were used in each of the three allocated locations.

### 3.1. Validity

There was acceptable agreement (<5% difference) between DO and activPAL4^TM^ sitting and stepping times during the standardised activities (Table 3). However, standing time was higher (+10.4%) for the activPAL4^TM^ than DO.

During normal speed walking, the activPAL4^TM^ step count was within acceptable agreement levels to DO forward purposeful step count. However, when all steps, or purposeful forward and backward steps included together, the activPAL4^TM^ demonstrated higher levels of disagreement with DO.

The activPAL4^TM^ underestimated the step count during fast walking/jogging compared to DO by approximately 20%.

During non-standardised activities, the activPAL4^TM^ step count was on average 10% higher than DO forward purposeful step count. When backward steps were added, the overall agreement of activPAL4^TM^ and DO was within acceptable limits. ActivPAL4^TM^ considerably underestimated step count compared to DO ‘all steps’ count (−30%).

Overall, there was considerable variation in agreement between activPAL4^TM^ step counts between individuals, especially within the non-standardised activities.

Second-by-second agreement for time allocated to sitting, standing and stepping was excellent (98.6%) (Table 4). Similarly, sensitivity and PPV values demonstrated high levels of validity. The lowest value achieved was 88.6% PPV for standing, supporting the observation of higher standing time in the activPAL4^TM^ than DO.

### 3.2. Reliability

Generally, the reliability of the activPAL4^TM^ was similar to that of the DO (Table 5). Across participants, average agreement was 98.2% for posture allocation across activPAL4^TM^ compared to 99.1% for DO. Similar excellent levels of reliability (ICC) were demonstrated for step count across both standardised and non-standardised activities for the activPAL4^TM^. Of note was a lower reliability for step count by DO during non-standardised activities (ICC(2, 1) 0.851–0.944).

## 4. Discussion

There have been previous publications suggesting that the activPAL^TM^ family of monitors demonstrate high levels of validity in measuring standardised activities with high levels of reliability [9,11,16,17]. There is some evidence that for non-standardised activities and faster walking/jogging pace that the monitor does not perform as well in comparison to DO. The activPAL4^TM^ iteration of the monitor has been presented as a new device and it is important to establish if this version has acceptable levels of validity and reliability under conditions in which it might be typically used. Children’s diverse movement patterns pose particular challenges to activity classification. For these reasons, this study was carried out to assess the validity and reliability of the activPAL4^TM^ in children 6–12 years of age. Generally, for standardised activities, the monitor performed very well for the detection of sitting and stepping time and purposeful forward-stepping activity. In comparison to direct observation, performance appears to be poorer in detecting faster steps and when activities become non-standard.

### 4.1. The Protocol

Activity protocols used in the evaluation of validity and reliability of activity monitors typically consist of a standardised and a non-standardised component [6,7]. The non-standardised section is often aimed at recreating more naturalistic movement patterns that may be present in the lives of the participants. For children aged 6–12 years old, it might be expected that they undertake more varied movement patterns than older children, with shorter periods in one posture and possibly more varied postures. The optimal conditions under which monitors should be assessed are free-living conditions where participants perform their normal movement patterns (e.g., preschool children [18]). However, this is very time consuming and as only a limited observation period is used, still does not include all movement patterns that the participants might engage in in real-world contexts. The non-standardised activity protocol used in this study was implemented to provide a range of self-selected stepping activity types, including a wide range of speeds, step lengths and directions of stepping. Thus, the non-standardised protocol should have provided a robust test of the activPAL4^TM^’s validity in counting steps against DO. The volume of stepping activity characterised through DO (Table 3) provides insight into the nature of the movements performed with a mean of 266 purposeful forward steps, but over 400 foot movements were recorded in the ‘all steps’ count. This suggests that there were many relatively small foot movements. The specific activities (‘keep up the balloon’, ‘throw the hoop over the post’, ‘musical chairs’) were used as they provided the opportunity for a range of movement patterns to fully test the validity of the activPAL4^TM^ against DO.

Evaluation of the validity of activity monitors requires a reference standard against which the monitor output can be compared. It is typical that direct observation is used as the gold standard reference in such cases. Results of the current study, established using three raters for DO, indicate that DO is a reliable method of establishing the reference standard for identification of sitting, standing and stepping time and steps during standardised activities. However, for non-standardised activities step count reliability was less good (e.g., ICC(2, 1) = 0.862 for forward purposeful steps). This suggests that even with a standardised definition of what constitutes a step, it was not possible for observers to consistently characterise the activity of 6–12-year-olds.

Children responded to instructions with varying levels of urgency and compliance. Participants all completed all sections of the protocol but at points had to be encouraged and reminded of the parameters of the particular activity. For example, speed of movement varied within tasks for some children.

### 4.2. Time of Activities

The outcomes of a validation and reliability study must be considered in the context of the proportion of time spent in each activity. The importance of this consideration is highlighted by the observed 10% higher standing time recorded by the activPAL4^TM^ during standardised activities than DO (Table 3). On closer inspection of the allocation of time within the activPAL4^TM^, it is revealed that following each transition from sitting to standing that the activPAL4^TM^ records a short time of standing time before it records stepping activity. However, under DO no such standing period was observed and sitting straight to stepping was often recorded. If stepping periods are relatively short, this activPAL4^TM^ designated standing period will become a large proportion of the related stepping period as assessed using DO. Thus, while a 10% difference in standing time was recorded for the protocol implemented in this study, if longer periods of stepping activity had been used, this proportion would most likely have been lower.

### 4.3. Validity

Judged against the a priori standard of 5% difference, standardised sitting, stepping and normal speed walking, forward purposeful steps were acceptably quantified by the activPAL4^TM^. High levels of sensitivity and PPV (Table 4) for time in sitting, standing and stepping suggest that the monitor is excellent at detecting postures, with the possible shortcoming of detecting more standing time compared to DO (PPV = 88.6%). Similar overestimation of standing time has been reported for the original activPAL^TM^ in 9–12-year-old children [19]; however, it was unclear if this was related to seat perching classification. This has previously been highlighted as a difficulty with DO characterisation of activity (original activPAL^TM^ [20], activPAL3^TM^ [11]). In comparison, a study on 4–6-year-olds [21] showed that the original activPAL^TM^ overestimated time spent sitting/lying by 5.9% and standing by 14.8%, consequently underestimated stepping by 10%. Researchers and clinicians must be aware of these limitations when interpreting outcomes from the activPAL4^TM^.

The sensitivity results presented in the current study appear to be superior to those previously reported in 5–12-year-old children for the activPAL3^TM^ [22]. Van loo et al. [22] reported sensitivity between 82.5% and 86.6% for time allocation to postures. This difference may be due to protocol differences.

From the step count results (Table 3) it appeared that the activPAL4^TM^ step count matched the forward purpose step volume recorded by DO better than the ‘all steps’ count or combined forward and backward step count. This suggests that the activPAL4^TM^ provides a valid measure of step count for purposeful stepping activity and does not include smaller foot movements as step counts. The excellent validity of the activPAL4^TM^ step count does not appear to extend to fast walking/jogging as overall these steps were underestimated by 20%. The reduction in step count for faster movements has been reported for previous versions of the monitor. For example, Aminian and Hinckson [23] reported data collected on 9–10-year-old children for fast walking and running with correlations of only r = 0.21–0.46 for observed vs. the original activPAL^TM^ step count. Sellers et al. [11] also highlighted reduced performance in step count for jogging activity with undercounting of 30% in young people (12 ± 4.1 years old) for the activPAL3^TM^ monitor.

Further insight into which steps are counted by the activPAL4^TM^ is gained from the non-standardised activities where the best agreement was recorded by combining forwards and backward purposeful steps (relatively large foot movements). The ‘all steps’ count recorded by DO was considerably higher (30%) than the activPAL4^TM^ count, reinforcing the observation that the activPAL4^TM^ does not count all foot movements (especially slower or shorter steps, e.g., activPAL3^TM^ [24,25,26,27].

### 4.4. Reliability

Differences in the exact internal configuration of activity monitors may affect interpretation of movement patterns. If, for example, the accelerometers within the devices are aligned in slightly different ways, this may result in changes in timing and type of activity classification. Assessing reliability is therefore important. Categorisation to sitting, standing and stepping times showed agreement for over 98% of the time (Table 4). Step count demonstrated excellent levels of reliability (ICC(1, 1) > 0.976). These outcomes suggest, even with slightly different placement of monitors on the thigh, that the outcomes from different activPAL4^TM^ devices show very high levels of agreement. These outcomes align with previously reported data for the activPAL3^TM^ where ICC(1, 1) values of 0.86 and above have been reported for posture classification and step count in young people [11].

As part of the evaluation of methods performed in this study, the reliability of observers was also assessed. The children who took part in this study were able to choose how they moved within each activity. This led to a range of different sitting postures and stepping movement patterns. It was challenging to be able to reliably characterise the movements using direct observation. This was particularly the case for stepping activity during the non-standardised activities. The lower level of reliability and the variation of this reliability between participants (Table 5) highlights a limitation of establishing the reference standard against which the activity monitor outcomes are compared. Therefore, there is a need for further research to be conducted on the performance of activPAL4^TM^ in different real-life conditions.

### 4.5. Limitations

Only a relatively small sample of participants was recruited to this study due to the extensive manual data analysis required in DO video assessment. However, the sample was sufficient to include a wide range of movement and stepping types within both standardised and non-standardised activity protocols, thus providing a suitably rigorous assessment of validity and reliability of the activPAL4^TM^.

To establish the validity and reliability of the activPAL4^TM^ under real-life conditions, it would have been necessary to study real-life scenarios. It is acknowledged that the standardised and non-standardised activities chosen within this study can only provide evidence of validity and reliability for these specific activities. However, by selecting activities that required a wide range of movements and stepping types, it was hoped that characteristics of validity and reliability would be relevant to real-world contexts.

For the assessment of reliability and validity of step detection, it is necessary to define what constitutes a step. As different step definitions will result in different step counts, the outcomes presented in this study must be considered in relation to the specific step definitions used. There is scope for additional work to explore how different step definitions influence outcomes of validity and reliability for the activPAL4^TM^.

To establish the between-activPAL4^TM^ (inter-device) reliability, activPAL4^TM^s were allocated to the three positions on the leg in an arbitrary way. To improve the assessment of reliability, this could have been carried out randomly. However, a range of activPAL4^TM^ devices were placed in each of the locations across participants, providing a suitable assessment of inter-device reliability.

The scope of the current study was limited to establishing the validity and reliability of the activPAL4TM in 6–12-year-old children. Further work is required to establish concurrent validity with previous versions of the monitor and to establish validity and reliability in other populations, e.g., adults or people with movement disorders.

## 5. Conclusions

The activPAL4^TM^ provided high validity against direct observation for detection of sitting and stepping time but slightly overestimated standing time. Purposeful forward stepping was detected with a high degree of accuracy for normal walking speeds, but steps were underestimated for faster walking/jogging. The characterisation of non-standardised activity stepping was challenging for DO. In general, it appeared that the activPAL4^TM^ recorded only larger stepping movements (forward and backwards) as steps but did not record smaller movements as steps. The activPAL4^TM^ demonstrated a high level of reliability between monitors as good and in some cases better than DO.

Overall, the activPAL4^TM^ appears to perform to a similar standard to earlier versions of the activPAL^TM^ device, recording standardised activities well but underestimating faster stepping. Further work is required to establish the exact nature of steps counted by the activPAL4^TM^ under non-standardised activity protocols.

## Figures and Tables

**Table 1:** Standardised and non-standardised activities in order of performance (all participants).

Standardised Activities	Non-Standardised Activities
Sitting (2 min) Standing (1 min): Quiet standing without stepping. Sitting (1 min) Walking with preferred stepping speed (1 min) Sitting (1 min) Fast walking/Jogging (1 min): Sitting (1 min) Sitting and playing a game on an iPad (2 min) Sitting and drawing (2 min)	Keep up the balloon (2 min): Participants were given a balloon, which they were instructed to keep up in the air by hitting it. They were allowed to move around the laboratory area. Throwing hoops over a post (2 min): Participants stood and threw hoops over a post between 1 and 4 m away. When all hoops had been thrown, they were retrieved and then the process was repeated until the end of the time. Musical chairs (2 min): Participants walked/jogged around two chairs facing outwards, approximately 1 m apart until the music stopped playing when they were required to sit down as quickly as possible in one of the chairs. The process was repeated until the end of the time.

**Table 2:** Definitions of posture and stepping used to interpret the video record.

Category	Definition
Posture	
Sitting	Gluteal region in contact with the seat and weight being taken by the seat.
Sit to stand transition	Mid-time point between being seated and standing.
Standing	Weight bearing through the feet in an upright posture. Leaning against a chair was classified as standing if weight was taken through the feet rather than the chair
Squatting	Gluteal region in contact with the heels was placed in an ‘unclassified posture’ category and the time was removed from the data analysis.
Stepping	
Purposeful forward step	Foot was unweighted and moved to a location in the forward direction with the heel of the foot landing in front of the toe of the contralateral foot. OR The trailing foot was unweighted and moved forward to align with the contralateral foot, as in the end of a forward-stepping bout. In all cases, the foot had to land outside the footprint of its original position.
Purposeful backward step	Foot was unweighted and moved from a position in front of or adjacent to the contralateral foot in the backward direction to end with the toe behind the heel of the contralateral foot. OR Foot was unweighted and moved from a position in front of the contralateral foot to a position adjacent to the contralateral foot, as in the end of a backward-stepping bout. In all cases, the foot had to land outside the footprint of its original position.
All steps	Foot was unweighted and placed in a different location. Movements forwards, backwards or sideways were allowed. The shoe must not land in exactly the same footprint as it started. Very small foot movements with ‘no meaningful change in position’ were not counted. Pivoting movements while maintaining contact with the floor were not counted.

**Table 3:** Direct observation (DO) mean (SD) values of activity categorisation (sitting, standing, stepping) and step count with Bland–Altman percentage mean differences between DO and activPAL4^TM^.

	Standardised Activities		
Activity categorisation	DO total duration (s) (mean ± SD)	activPAL4^TM^ total duration (s) (mean ± SD)	Percentage mean difference (LLOA, ULOA) (%) *
Sitting	539.5 (6.1)	538.5 (6.2)	−0.2 (−0.9, 0.4)
Standing	65.0 (4.1)	71.6 (3.7)	10.4 (1.2, 19.7)
Stepping	120.5 (6.3)	115.6 (6.0)	−4.0 (−10.8, 2.7)
Step type	DO step count (mean (SD))	activPAL4 ^TM^ step count (mean (SD))	Percentage mean difference (LLOA, ULOA) (%)
Normal walking			
Forward purposeful	96 (27)		−2.9 (−21.2, 15.5)
All	102 (22)	91 (21)	−10.5 (−36.2, 15.2)
Forward + backward purposeful	98 (22)		−7.5 (−27.7, 12.6)
Fast walking/jogging			
Forward purposeful	152 (23)		−19.3 (−50.7, 12.1)
All	158 (21)	120 (16)	−22.6 (−53.1, 8.0)
Forward + backward purposeful	152 (23)		−19.4 (−50.8, 12.0)
	Non-standardised activities		
Step type	DO step count (mean (SD))	activPAL4 ^TM^ step count (mean (SD))	Percentage mean difference (LLOA, ULOA) (%)
Forward purposeful	266 (48)		10.0 (−25.7, 45.6)
All	419 (72)	292 (68)	−30.0 (−53.0, −7.1)
Forward + backward purposeful	293 (51)		−0.3 (−33.5, 32.9)

* Calculated as (activPAL4—DO)/DO as a %. LLOA (lower) and ULOA (upper) limits of agreement.

**Table 4:** Standardised activities second-by-second posture agreement, sensitivity and positive predictive value (PPV): DO and activPAL4^TM^.

Activity Categorisation		
Sitting/standing/stepping	Agreement (%) 98.6 (0.6) (97.2, 99.3)	
	Sensitivity (%)	PPV (%)
Sitting	99.7 (0.3) (98.9, 99.9)	99.9 (0.1) (99.5, 100.0)
Standing	97.8 (3.8) (86.7, 100.0)	88.6 (3.0) (82.4, 93.8)
Stepping	94.2 (2.2) (88.7, 98.1)	98.2 (2.8) (91.6, 100.0)

Mean (SD) (range), with reference as DO.

**Table 5:** Reliability of DO and activPAL4^TM^ outcomes.

activPAL4^TM^ (3 activPAL4^TM^s)	
Time allocation to sitting/standing/stepping	Agreement (%)
Standardised activities	98.2 (3.3) (87.7–99.7)
Step count	Reliability (ICC(1, 1))
Normal walking	0.994 (0.986, 0.998)
Fast walking/jogging	0.976 (0.940, 0.992)
Non-standardised activities	0.983 (0.957, 0.994)
Direct observation (3 observers)	
Time allocation to sitting/standing/stepping	Agreement (%)
Standardised activities	99.1 (0.3) (98.6–99.6)
Step count	Reliability (ICC(2, 1))
Normal walking	
Forward Purposeful	0.997 (0.993, 0.999)
All	0.997 (0.993, 0.999)
Forward + Backward purposeful	0.997 (0.992, 0.999)
Fast walking/jogging	
Forward Purposeful	0.986 (0.965, 0.995)
All	0.995 (0.984, 0.998)
Forward + Backward purposeful	0.986 (0.964, 0.995)
Non-standardised activities	
Forward Purposeful	0.862 (0.418, 0.961)
All	0.944 (0.811, 0.983)
Forward + Backward purposeful	0.851 (0.389, 0.958)

Agreement: Mean (SD) (range), ICC: 95% confidence interval.

## Data Availability

To enquire about access to the data used to develop outcome in this paper please contact the lead author.
